# TTK is a favorable prognostic biomarker for triple-negative breast cancer survival

**DOI:** 10.18632/oncotarget.13245

**Published:** 2016-11-09

**Authors:** Qianqian Xu, Yali Xu, Bo Pan, Liangcai Wu, Xinyu Ren, Yidong Zhou, Feng Mao, Yan Lin, Jinghong Guan, Songjie Shen, Xiaohui Zhang, Changjun Wang, Ying Zhong, Liangrui Zhou, Zhiyong Liang, Haitao Zhao, Qiang Sun

**Affiliations:** ^1^ Department of Breast Surgery, Peking Union Medical College Hospital, Chinese Academy of Medical Sciences and Peking Union Medical College, Beijing, China; ^2^ Department of Liver Surgery, Peking Union Medical College Hospital, Chinese Academy of Medical Sciences and Peking Union Medical College, Beijing, China; ^3^ Department of Pathology, Peking Union Medical College Hospital, Chinese Academy of Medical Sciences and Peking Union Medical College, Beijing, China

**Keywords:** triple-negative breast cancer (TNBC), TTK, biomarker, prognostic indicator, survival analysis

## Abstract

**Purpose:**

Previous studies demonstrate that threonine and tyrosine kinase (TTK) is overexpressed in triple-negative breast cancer (TNBC), but there are conflicting results regarding its effect on TNBC survival. The purpose of this study was to assess the prognostic significance of TTK expression in primary TNBC.

**Results:**

Of 169 consecutive cases eligible for this study, 164 included follow-up information. Cytoplasm and membrane TTK staining was observed in 99.4% of cases, while 5.9% displayed whole cell immunostaining. At a discriminating threshold of 55, elevated TTK expression was associated with prolonged disease free survival (DFS) (*p* < 0.001) and overall survival (OS) (*p* = 0.024) in primary TNBC and prolonged DFS in individual basal-like (*p* = 0.001) and non-basal-like (*p* = 0.001) TNBC subtypes. In addition, Cox regression analysis demonstrated that elevated TTK expression was an independent prognostic factor for DFS in TNBC (*p* < 0.001).

**Methods:**

TTK expression of 169 samples was tested by immunohistochemistry (IHC). A receiver operating characteristic (ROC) curve was used to identify a cutpoint for TTK expression, which was analyzed for its association with patients' clinicopathological factors and survival using Chi-square, log-rank, and Cox regression analyses.

**Conclusions:**

TTK is a favorable prognostic biomarker associated with TNBC survival.

## INTRODUCTION

Breast cancer is the most common cancer and the leading cause of cancer-related death in women worldwide [1]. It consists of the luminal A, luminal B (HER2-negative), luminal B (HER2-positive), HER2-positive (non-luminal), and triple-negative (ductal) subtypes. Approximately 15% of invasive breast cancers are triple-negative breast cancers (TNBC) that lack estrogen receptor (ER), progesterone receptor (PR), and human epidermal growth factor receptor 2 (HER2) expressions and usually exhibit a high pathological grade and more aggressive clinical behavior. Currently, chemotherapy is the only systemic treatment modality for TNBC patients.

The spindle assembly checkpoint (SAC) is a signaling cascade that prevents chromosome missegregation by arresting mitosis until all chromosomes are properly attached to the mitotic spindle [2]. As the core SAC kinase, TTK kinase is a dual-specificity kinase able to phosphorylate serine/threonine and tyrosine residues [3], and critical for the recruitment of SAC proteins to unattached kinetochores, mitotic checkpoint complex (MCC) formation, and mitotic arrest [4]. Thus, the inhibition of TTK activity causes cells to prematurely exit mitosis with unattached chromosomes, resulting in severe chromosome missegregation, aneuploidy, and eventually cell death [5–8]. The increased expression of mitotic checkpoint genes contributes to chromosomal instability in cancer cells [9–12]. Elevated TTK mRNA levels are found in several human cancers, including thyroid carcinoma, breast cancer, lung cancer, pancreatic cancer, prostate cancer, and melanoma, as well as glioblastoma and hepatocellular carcinoma—where it is associated with poor prognosis [9, 11, 13-18].

Previous studies show that TTK is overexpressed in breast cancer tissue and cells, particularly in the HER2-positive and TNBC subtypes [9, 10, 19, 20]. Whether it is also a prognostic factor in TNBC remains disputed. In the current study, we retrospectively analyzed TTK expression in 169 TNBC samples and investigated the correlation between TTK expression and TNBC prognosis.

## RESULTS

### Clinicopathological characteristics and survival data of the cohort

The present study enrolled 169 consecutive TNBC cases (Table 1). The median age of patients at surgery was 51 years (range, 16–81 years). Most cases were immediate- to high-grade invasive breast ductal carcinoma (162/169, 95.5%), and 91.7% (155/169) of patients received a modified radical mastectomy. Of cases with available adjuvant treatment information, 99.3% (134/135) received chemotherapy (one ceased treatment because of an allergic reaction) and 34.3% (48/140) received radiation. Five cases lost follow-up. The median overall follow-up period was 1864 days (range, 1104–2373 days), and the five-year DFS and OS rates were 76.2% and 90.6%, respectively.

**Table 1:** Baseline clinicopathological characteristics and treatments of the cohort

Characteristics (n=169)	Values
**Age at diagnosis:**	
Median, range (years)	51 (26~81)
<40	30 (17.8%)
40~59	103 (60.9%)
≥60	36 (21.3%)
**surgery**	
MRM	155 (91.7%)
BCS	14 (8.3%)
**Histology subtype**	
IDC	162 (95.9%)
others	7 (4.1%)
**Grade**	
Low	1 (0.6%)
Intermediate	43 (25.4%)
High	125 (74%)
**Lymphovascular invasion**	
No	156 (92.3%)
Yes	13 (7.7%)
**Tumor size, cm**	
≤2	82 (48.5%)
2~5	80 (47.3%)
>5	7 (4.1%)
**Number of positive lymph nodes**	
0	89 (52.7%)
1~3	36 (21.3%)
4~9	21 (12.4%)
≥10 or 3^rd^ stop metastasis	23 (13.6%)
**Pathologic stage**	
I	49 (29.0%)
II	73 (43.2%)
III	47 (27.8%)
**Ki67**	
<20%	16 (9.5%)
≥20%	153 (90.5%)
**P53 status**	
Negative	75 (44.4%)
Positive	94 (55.6%)
**Molecular subtype**	
Basal-like TNBC	97 (57.4%)
Non basal-like TNBC	38 (22.5%)
NA	34 (20.1%)
**Chemotherapy**	
None*	1 (0.6%)
A-based	34 (20.1%)
T-based	21 (12.4%)
AT-based	67 (39.6%)
Others	13 (7.7%)
NA	34 (20.1%)
**Radiation**	
No	92 (54.4%)
Yes	48 (28.4%)
NA	29 (17.2%)
**Follow-up**	
Median, range (days)	1864 (1104~2373)
5-y disease free rate (%)	76.2
5-y survival rate (%)	90.6

* One ceased chemotherapy because of an allergic reaction

Abbreviations: MRM modified radical mastectomy, BCS breast-conserving surgery, IDC invasive ductal carcinoma, NA not available, A anthrocycline, T taxanes.

### TTK expression and cutpoint determination

**Figure 1:**
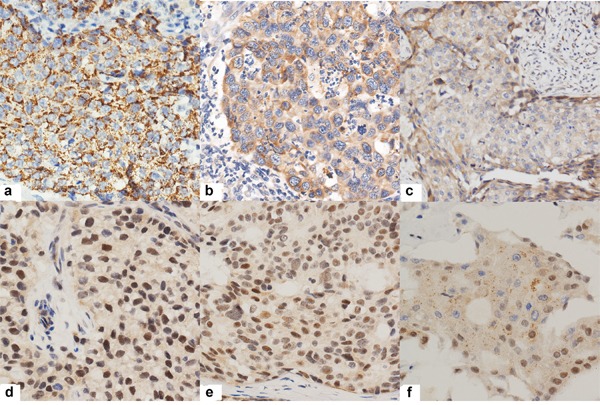
Representative immunohistochemical results of TTK positive tumor cells **a.**, **b.**, **c.** showing TTK cytoplasm and membrane positivity with 3+, 2+ and 1+ intensity respectively. **d.**, **e.**, **f.** showing nucleus positivity with 3+, 2+ and 1+ intensity.

**Figure 2:**
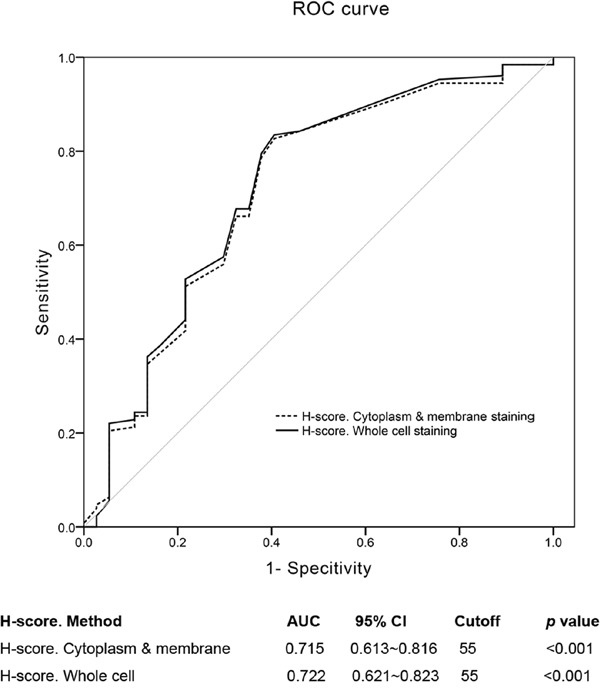
Cutoff values of “H-score. Cytoplasm & membrane” and “H-score. Whole cell” ROC curves showed that the cutpoint of the two methods were both 55. The “H-score. Whole cell” assessment method had a little higher AUC (0.722 (0.613~0.816)) than the former method (0.715 (0.621~0.823)).

TTK expression was analyzed in 169 patients. Most samples (168/169, 99.4%) displayed cytoplasm and membrane staining, of which 10 cases (5.9%) had concomitant nuclear expression (Table 2, Figure 1). Both “H-score. Cytoplasm & membrane” and “H-score. Whole cell” methods yielded a cutoff value of 55 with nearly identical *p* values (*p* < 0.001, Figure 2); however, the area under the curve (AUC) was slightly higher in the whole cell staining analysis, thus this scoring method was selected and the discriminating threshold set at 55.

**Table 2:** TTK expression results

	Positive cells rate (%) Median (range)	Number of cases (%)
**Cytoplasm & membrane only staining**		
Negative	0	1 (0.6%)
Positive	90 (5~220)	168 (99.4%)
1+	40 (5~90)	165 (97.6%)
2+	20 (5~80)	123 (72.8%)
3+	7.5 (5~40)	32 (18.9%)
**Nucleus staining**		
Negative	0	159 (94.1%)
Positive	40 (5~160)	10 (5.9%)
1+	25 (5~40)	6 (3.6%)
2+	80 (30~80)	4 (2.4%)
3+	10	1 (0.6%)
**Whole cell staining**		
Negative	0	1 (0.6%)
Positive	90 (5~340)	168 (99.4%)

### Correlation between TTK and clinicopathological factors

The association between clinicopathological characteristics and TTK expression are summarized in Table 3. No significant association was found between TTK status and age, histology subtype, grade, lymphovascular invasion (LVI), tumor size, number of positive lymph nodes, pathologic stage, Ki67 index, and p53 status; however, TTK overexpression was significantly higher in the basal-like TNBC subgroup.

**Table 3:** Correlations between TTK expression and clinicopathologic characteristics

characteristics	TTK (n=166)		X^2^	*p*
	Low expression	High expression		
**Age at diagnosis: 51 (26~81)**			**2.415**	**0.299**
<40	9 (5.3%)	21 (12.4%)		
40~59	22 (13.0%)	81 (47.9%)		
≥60	12 (7.1%)	24 (14.2%)		
**Histology subtype**			**1.289**	**0.256**
IDC	43 (25.4%)	119 (70.4%)		
others	0 (0%)	7 (4.1%)		
**Grade**			**0.006**	**0.937**
Low/Intermediate	11 (6.5%)	33 (19.5%)		
High	32 (18.9%)	93 (55.0%)		
**Lymphovascular invasion**			**2.111**	**0.146**
No	37 (21.9%)	119 (70.4%)		
Yes	6 (3.6%)	7 (4.1%)		
**Tumor size (cm)**			**2.841**	**0.242**
≤2	17 (10.1%)	65 (38.5%)		
2~5	25 (14.8%)	55 (32.5%)		
>5	1 (0.6%)	6 (3.6%)		
**Number of positive lymph nodes**			**3.761**	**0.288**
0	18 (10.8%)	71 (42.8%)		
1~3	10 (6.0%)	26 (15.7%)		
4~9	6 (3.0%)	15 (9.0%)		
≥10 or 3^rd^ stop metastasis	9 (4.8%)	14 (7.8%)		
**Pathologic stage**			**2.344**	**0.310**
I	9 (5.3%)	40 (23.7%)		
II	19 (11.2%)	54 (32.0%)		
III	15 (8.9%)	32 (18.9%)		
**Ki67 index**			**0.743**	**0.389**
<20%	6 (3.6%)	10 (6.0%)		
≥20%	37 (21.1%)	116 (69.3%)		
**P53 status**			**0.001**	**0.977**
Negative	19 (11.2%)	56 (33.1%)		
Positive	24 (14.2%)	70 (41.4%)		
**Molecular subtype**			**3.870**	**0.049**
Basal-like TNBC	22 (13.0%)	75 (44.4%)		
Non basal-like TNBC	15 (8.8%)	23 (13.6%)		
NA	6 (3.6%)	28 (16.6%)		

Abbreviations: IDC invasive ductal carcinoma, A Anthracyclin, T Taxanes, NA Not available.

### Survival analysis

**Figure 3:**
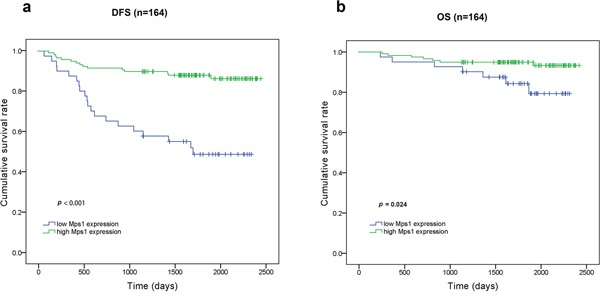
Kaplan-Meier curves for DFS and overall survival OS according to TTK expression **a.** TTK^high^ patients had a longer DFS than TTK^low^ patients (*p*<0.001). **b.** TTK^high^ patients had a longer OS than TTK^low^ patients (*p*=0.024).

Clinical follow-up data were available for 164 out of 169 patients. Univariate survival analysis revealed that LVI, increased tumor size, positive lymph nodes, advanced stage, specific chemotherapy regimen (Anthracycline+Taxanes (AT)-based regimen), and lower TTK expression were associated with shorter DFS (Table 4). All of these factors excepting increased tumor size, as well as radiation therapy, were also associated with a reduced OS (Table 4). The Kaplan-Meier curves for DFS and OS against TTK expression are shown in Figure 3. Conversely, age, surgery type, histology subtype, grade, p53 status, Ki67 index, and molecular subtype had no impact on survival in our study.

**Table 4:** Univariate analyses of survival against various characteristics

Variables	No.Pat (n=164)	DFS			OS		
		No.even (n=37)	X^2^	P	No.evetnt (n=15)	X^2^	P
**Age**			**4.316**	**0.116**		**2.232**	**0.328**
<40	30	20			3		
40~59	100	17			7		
≥60	34	10			5		
**Surgery**			**1.970**	**0.160**		**0.102**	**0.749**
MRM	150	36			14		
BCS	14	1			1		
**Histology**			**0.017**	**0.895**		**0.815**	**0.367**
IDC	159	36			14		
others	5	1			1		
**Grade**			**0.402**	**0.526**		**0.244**	**0.621**
Low/Intermediate	42	11			3		
High	122	26			12		
**LVI**			**22.276**	**<0.001**		**3.864**	**0.049**
No	151	29			12		
yes	13	8			3		
**P53**			**0.289**	**0.591**		**0.022**	**0.883**
Neg	73	15			7		
Pos	91	22			8		
**Ki67 index**			**1.226**	**0.268**		**2.963**	**0.085**
<20%	14	5			3		
≥20%	150	32			12		
**Tumor size**			**14.300**	**0.001**		**1.858**	**0.395**
≤2cm	80	11			5		
2-5cm	78	22			9		
>5cm	6	4			1		
**Number of positive lymph nodes**			**32.757**	**<0.001**		**23.720**	**<0.001**
0	89	11			4		
1~3	35	9			2		
4~9	20	5			2		
≥10 or 3^rd^ stop metastasis	20	12			7		
**Stage**			**18.048**	**<0.001**		**11.361**	**0.003**
I	49	4			2		
II	73	15			4		
III	42	18			9		
**Molecular subtype**			**0.206**	**0.650**		**0.295**	**0.587**
Basal-like TNBC	96	22			9		
Non basal-like TNBC	37	9			2		
NA	31	6			4		
**Chemotherapy**			**10.014**	**0.018**		**11.365**	**0.010**
None*	1	0			0		
A-based	34	4			0		
T-based	21	3			2		
AT-based	67	20			6		
Others	12	6			4		
NA	29	4			3		
**Radiation**			**1.299**	**0.254**		3.848	**0.050**
No	91	20			6		
Yes	48	14			8		
NA	35	3			1		
**TTK expression**			**29.438**	**<0.001**		**6.653**	**0.010**
<55	43	22			7		
≥55	121	15			7		

* One ceased chemotherapy because of an allergic reaction.

Abbreviations: MRM modified radical mastectomy, BCS breast-conserving surgery, IDC invasive ductal carcinoma, NA not available, A anthrocycline, T taxanes.

Cox regression multivariate analysis of DFS and OS revealed that TTK expression was an independent predictor for DFS (*p* < 0.001), but not for OS, whereas the number of positive lymph nodes was an independent predictor for both DFS (*p* < 0.001) and OS (*p* = 0.005) (Table 5). Cox regression analysis results for other clinical variables are shown in Supplemental Table S1–S3.

**Table 5:** Multivariate analysis of survival against various characteristics

	DFS			OS		
	Hazard ratio	95.0% confidence interval	*p* value	Hazard ratio	95.0% confidence interval	*p* value
**Age**			**0.268**			**0.331**
<40	1			1		
40~59	0.532	0.228~1.240		0.627	0.134~2.929	
≥60	0.862	0.321~2.310		1.715	0.341~8.617	
**surgery**			**0.080**			**0.861**
MRM	1			1		
BCS	0.157	0.020~1.243		1.223	0.128~11.640	
**Histology subtype**			**0.626**			**0.415**
IDC	1			1		
Others	1.691	0.205~13.946		2.540	0.270~23.874	**0.186**
**Grade**			**0.601**			
Low/Intermediate	1			1		
High	0.815	0.379~1.753		2.632	0.627~11.041	
**P53**			**0.857**			**0.392**
Negative	1			1		
Positive	1.067	0.526~2.164		0.597	0.183~1.947	
**Ki67**			**0.662**			**0.223**
<20%	1			1		
≥20%	1.256	0.453~3.483		0.391	0.086~1.769	
Number of positive lymph nodes*			**0.000**			**0.002**
0	1			1		
1~3	2.256	0.915~5.561		1.244	0.216~7.167	
4~9	1.661	0.569~4.851		1.592	0.273~9.276	
≥10 or 3^rd^ stop metastasis	7.195	2.965~17.459		10.690	2.814~40.611	
**TTK expression**			**0.000**			**0.111**
<55	1			1		
≥55	0.197	0.098~0.398		0.394	0.125~1.240	

* Because of multicollinearity between lymphovascular invasion, tumor size, number of positive lymph nodes, and pathological stage, only number of positive lymph nodes entered Cox regression model in this table.

Abbreviations: MRM modified radical mastectomy, BCS breast-conserving surgery, IDC invasive ductal carcinoma.

**Figure 4:**
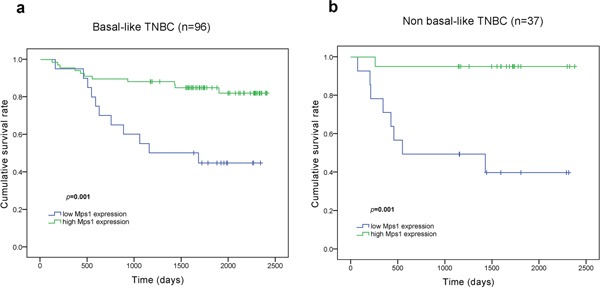
Kaplan-Meier curves for DFS according to TTK expression among TNBC subgroup corhorts **a.** TTK^high^ patients had a longer DFS than TTK^low^ patients among basal-like TNBC cohort (*p*=0.001). **b.** TTK^high^ patients had a longer DFS than TTK^low^ patients among non basal-like TNBC cohort (*p*=0.001).

Further subgroup analysis by log-rank testing showed that TTK expression associated with DFS in both basal-like (*p* = 0.001) and non-basal-like (*p* = 0.001) TNBC cases (Figure 4).

## DISCUSSION

In the present study, it was found that TTK expression was associated with the TNBC molecular subtype and correlated with a better prognosis. Cox regression multivariate analysis confirmed TTK expression as an independent favorable prognostic indicator for TNBC. Consistent with our results, Maire et al [20] reported that low TTK mRNA expression in TNBC is significantly associated with a poorer overall survival, an increased risk of metastasis, and shorter DFS. However, AI-Ejeh et al [19] found that TTK protein levels were elevated specifically in highly aggressive tumors, leading to poor survival of less than 2 years. Both of the two studies had small sample sizes (39 and 69, respectively), and used different methods (RNA microarray analysis and IHC analysis) to determine expression. In addition, the latter study only used staining intensity to evaluate TTK protein expression, which might be far from a sufficient semi-quantitative assessment. Here, TTK protein expression was analyzed semi-quantitatively using the H-scoring system, which accounts for both staining intensity and the percentage of positive cells. A discriminating threshold for TTK expression was then determined with ROC curve analysis. This is the first study to evaluate the impact of TTK protein expression on the survival of a consecutive TNBC cohort with the largest sample size.

TTK has universally conserved functions at kinetochores to monitor the correct bipolar attachment and tension of all chromosomes to the mitotic spindle. Loss of TTK function causes chromosomal missegregation and induces cells apoptosis, while high level of TTK enable cells with higher aneuploidy to survive [9, 12, 16]. However, an auto-regulatory negative feedback loop between TTK and B-Raf^WT^/ERK signaling was found in melanoma cells [21]. Deregulation of B-Raf/ERK signaling pathway is frequently observed and plays a central role in the carcinogenesis and maintenance of several cancers [22]. The negative feedback loop might exist in TNBC cells and low level of TTK activates the B-Raf/ERK signaling, which contributes to the invasiveness of cancer cells and poor survival of patients. Therefore, TTK plays a special and complicated role in breast cancer and should be regarded as an important regulator factor.

The use of TTK expression as a positive prognostic indicator may help in personalized prognosis evaluation and treatment. Apart from the traditional prognostic indicators-ER, PR and HER2, assessment of the TTK expression provides additional prognostic information. For example, in patients with early breast cancer of hormone receptor positive and HER2 negative, a high level of TTK expression predicts a good survival and may spare adjuvant chemotherapy safely. However, a low level of TTK expression may hint the need of cytotoxic agents. The data also identified a low TTK-expression TNBC subset with significantly worse prognosis in both basal-like and non-basal-like tumors. The molecular subgroup which was ER^–^/PR^–^/HER2^–^/TTK^low^ presented a quadrate-negative phenotype and was defined as quadrate-negative breast cancer (QNBC) in the current study. In other words, the four-biomarker panel can identify some TNBC cases with dismal prognosis which might require more intense treatment than others.

Numerous studies suggest that TTK may be a promising drug target for anticancer therapy, and several small-molecule inhibitors targeting this kinase are currently under development [5–8, 23–25] or have entered the clinic (BAY 1161909, NCT02138812; BAY 1217389, NCT02366949) [26]. However, the patient groups that would benefit from TTK-targeted therapy remain unclear. Moreover, because TTK is expressed in all proliferating human cells [27], these inhibitors should be used cautiously to avoid severe adverse effects. TTK-567(SYRNEIAYL) epitope peptide was also used to elicit cytotoxic T lymphocytes to establish cancer vaccines in lung, esophageal and advanced biliary tract cancers [28, 29].

This study has several limitations, primarily its retrospective nature and single-institution sample. The lack of a standard method to assess TTK expression is also a disadvantage. In addition, multicollinearity existed between lymph-vascular invasion, tumor size, number of positive lymph nodes, and pathological stage in the clinicopathological data, but these characteristics were considered separately in the Cox regression model. The Cox regression analysis showed TTK expression not an independent impact factor for OS. It might contribute to the small number of deaths and relative short follow-up period. For these reasons, a multi-institution prospective study with all molecular subtypes will be required to verify the prognostic role of TTK and to further assess the clinical significance of QNBC group.

In conclusion, while these findings should be confirmed with a larger patient population, our results suggest that TTK expression is a favorable independent prognostic biomarker for TNBC survival.

## MATERIALS AND METHODS

### Patients and tumor specimens

**Figure 5:**
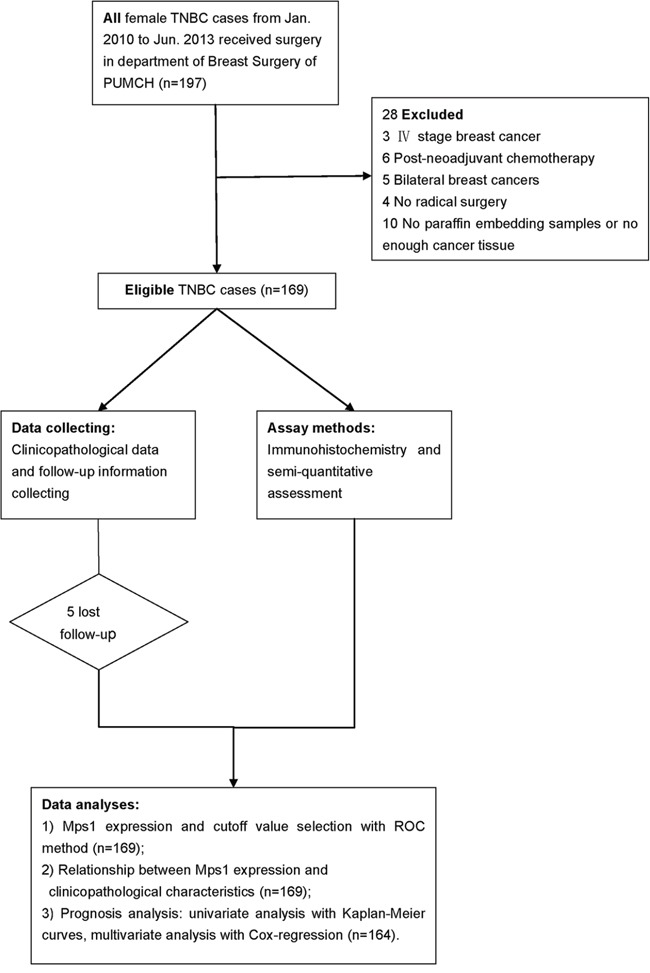
Flow diagram of the study Total 197 consecutive TNBC cases were reviewed and 169 were included in this study. After clinicopathological and follow-up data collecting and immunohistochemistry experiment, associations between TTK expression with clinicopathological factors and survival were analyzed.

The study population included patients with unilateral TNBC who received curative surgery and adjuvant treatment according to guidelines at the Department of Breast Surgery of Peking Union Medical College Hospital (PUMCH) between January 2010 and June 2013. Cases with insufficient paraffin-embedded tumor tissue or those treated with neoadjuvant therapy were excluded from the study, resulting in a total of 169 consecutive enrolled patients (Figure 5). The follow-up period lasted from the date of surgery until June 2016. The primary endpoint was Disease-free survival (DFS) and secondary endpoint was overall survival (OS). DFS and OS intervals were defined as the time from surgery to the date of breast cancer-related relapse or death, respectively. Relapsed disease and metastasis were verified by diagnostic imaging and pathology during follow-up. This study was approved by the Ethics Committee of PUMCH and informed consent was obtained from each patient.

### Immunohistochemistry staining and analysis

All tissues were fixed in 10% neutral-buffered formalin immediately after surgical resection and embedded in paraffin. Serial sections (3–4 μm) were mounted on adhesion slides. Immunohistochemical staining was performed with a Ventana Benchmark XT autostainer using standard autostaining protocols (Ventana Medical Systems Inc., Tucson, AZ) and all slides were processed according to the manufacturer's protocols. The antibodies used for biomarker expression analysis and their optimized staining conditions are described in Table 6. Positive and negative controls were performed using the manufacturer-recommended control tissue and isotype antibody respectively.

**Table 6:** Antibodies and optimizations for the immunohistochemical analysis

Antibody	Clone	Dilution	Source	Positive style	Positive control	Cutoff values (%)	Heat-induced antigen retrieval by 1 mM EDTA in 10 mM Tris buffer (pH 8.5)	Incubation
ERα	Rabbit monoclonal (EP1)	Prediluted	Epitomics	Nuclear staining	Breast cancer or human, endometrial carcinoma	≥1	100 °C, 30 min	37 °C, 32 min
PR	Rabbit monoclonal (EP2)	Prediluted	Epitomics	Nuclear staining	Breast cancer	≥1	100 °C, 30 min	37 °C, 32 min
Her-2	Rabbit monoclonal (4B5)	Prediluted	Ventana	Membrane staining	Breast cancer	According to [34]	100 °C, 30 min	37 °C, 32 min
CK5/6	Mouse monoclonal (D5/16 B4)	Prediluted	Dako	Membrane and/or cytoplasmic staining	Mesothelioma	≥5	100 °C, 30 min	37 °C, 32 min
EGFR	Rabbit monoclonal (5B7)	Prediluted	Ventana	Membrane and/or cytoplasmic staining	Skin	>25	100 °C, 30 min	37 °C, 32 min
P53	Mouse monoclonal (DO7)	Prediluted	MXB	Nuclear staining	Colon adenocarcinoma	≥5	100 °C, 30 min	37 °C, 32 min
Ki-67	Mouse monoclonal (MIB1)	Prediluted	ZSGB-BIO	Nuclear staining	Breast cancer	≥14	100 °C, 30 min	37 °C, 32 min
TTK	Rabbit polyclonal	1:100	Sigma	Cytoplasmic and/ or membrane staining, rare nuclear staining	Small intestine	≥55	100 °C, 30 min	37 °C, 32 min

IHC slides were evaluated by two experienced pathologists in a blinded manner. Positive staining was defined as cells with staining patterns specified in Table 6. H-scoring was used to quantify TTK staining because no uniform standard exists [30–34]. For this, the overall staining intensity (0-3) was multiplied by the percentage of positive cells (0-100%), and all values were added to generate a final H-score ranging from 0 to 300. For ROC curve analysis, TTK staining was scored based on staining only in the cytoplasm and membrane (“H-score. Cytoplasm & membrane”) or in the whole cell including cytoplasm, membrane and nucleus (“H-score. Whole cell”). The expression of other biomarkers was determined based on the cutoff value provided in Table 6. For HER-2 specifically, staining intensities were rated as (0), (1+), (2+), and (3+) according to the HER2 test guide for breast cancer [35]. HER-2 (0) or (1+) slides were categorized as HER-2-negative, while HER-2 (3+) slides were classified as HER-2-positive. HER-2 (2+) cases were subjected to secondary analysis by fluorescent in situ hybridization (FISH) to confirm HER-2 status on a genetic basis, and those determined to be negative were enrolled in the present study. The five-biomarker immunopanel (ER, PR, HER2, CK5/6, EGFR) was also used to classify TNBC cases as basal-like (ER–/PR–/HER2– with EGFR+ and/or CK5/6+) or non-basal-like (ER–/PR–/HER2–/EGFR–/CK5/6–).

### Statistical analysis

Statistical analysis was performed in SPSS 17.0 (SPSS, Chicago, IL, USA). Qualitative variables were compared with chi-square tests and univariate log-rank testing was used to assess the associations of DFS and OS with disease covariates to identify prognostic factors. Cox regression multivariate analysis was performed to determine the significance of prognostic factors. All *p* values were two sided and considered significant at α = 0.05.

Receiver operating characteristic (ROC) curve analysis was used to assess the discriminatory power of prognostic factors to identify the optimal value of a continuous variable to differentiate between a probability of survival and death [36, 37].

## SUPPLEMENTARY MATERIALS FIGURES AND TABLES


