# Supplementary material for: TTK is a favorable prognostic biomarker for triple-negative breast cancer survival
Source: Oncotarget. 2016 Nov 9;7(49):81815–29. doi: 10.18632/oncotarget.13245 (PMC5348432; doi:10.18632/oncotarget.13245)
Supplement: Supplementary file 1 [file oncotarget-07-81815-s001.pdf]

TTK is a favorable prognostic biomarker for triple-negative breast cancer survival

SUPPLEMENTARY TABLES

Supplementary Table S1: Multivariate analysis of survival against various characteristics

	DFS			OS		
	Hazard ratio	95.0% confidence interval	*p* value	Hazard ratio	95.0% confidence interval	*p* value
**Age**			**0.111**			**0.441**
<40	1			1		
40~59	0.413	0.180~0.946		0.538	0.127~2.289	
≥60	0.613	0.246~1.530		1.153	0.249~5.346	
**surgery**			**0.149**			**0.983**
MRM	1			1		
BCS	0.223	0.029~1.709		1.023	0.119~8.815	
**Histology subtype**			**0.425**			**0.274**
IDC	1			1		
Others	2.334	0.291~18.725		3.408	0.379~30.621	**0.507**
**Grade**			**0.118**			
Low/ Intermediate	1			1		
High	0.558	0.269~1.158		1.563	0.417~5.856	
**P53**			**0.465**			**0.848**
Negative	1			1		
Positive	1.289	0.653~2.546		0.904	0.319~2.557	
**Ki67**			**0.433**			**0.272**
<20%	1			1		
≥20%	1.534	0.526~4.474		0.450	0.108~1.868	
**Lymphovascular invasion**			**<0.001**			**0.229**
0	1			1		
1~3	5.835	2.379~14.310		2.325	0.587~9.210	
**TTK expression**			**<0.001**			**0.061**
<55	1			1		
≥55	0.199	0.100~0.399		0.343	0.112~1.020	

*Because of multicollinearity between LVI, tumor size, number of positive lymph nodes, and pathological stage, only lymphovascular invasion entered Cox regression model in this table.
Abbreviations: MRM modified radical mastectomy, BCS breast-conserving surgery, IDC invasive ductal carcinoma.

Supplementary Table S2: Multivariate analysis of survival against various characteristics

	DFS			OS		
	**Hazard ratio**	**95.0% confidence interval**	***p* value**	**Hazard ratio**	**95.0% confidence interval**	***p* value**
**Age**			**0.617**			**0.568**
<40	1			1		
40~59	0.659	0.281~1.550		0.646	0.148~2.815	
≥60	0.836	0.325~2.150		1.242	0.259~5.948	
**surgery**			**0.171**			**0.945**
MRM	1			1		
BCS	0.240	0.031~1.851		1.078	0.125~9.320	
**Histology subtype**			**0.751**			**0.302**
IDC	1			1		
Others	1.410	0.169~11.741		3.264	0.345~30.895	
**Grade**			**0.088**			**0.539**
Low/ Intermediate	1			1		
High	0.510	0.235~1.106		1.528	0.395~5.912	
**P53**			**0.776**			**0.831**
Negative	1			1		
Positive	1.104	0.559~2.180		0.892	0.314~2.534	
**Ki67**			**0.530**			**0.114**
<20%	1			1		
≥20%	0.729	0.272~1.956		0.321	0.078~1.312	
**Tumor size (cm)***			**0.001**			**0.480**
≤2	1			1		
2~5	1.941	0.911~4.135		1.792	0.571~5.625	
>5	11.727	3.111~44.205		3.139	0.316~31.143	
**TTK expression**			**<0.001**			**0.051**
<55	1			1		
≥55	0.168	0.082~0.346		0.331	0.109~1.003	

*Because of multicollinearity between lymphovascular invasion, tumor size, number of positive lymph nodes, and pathological stage, only tumor size entered Cox regression model in this table.
Abbreviations: MRM modified radical mastectomy, BCS breast-conserving surgery, IDC invasive ductal carcinoma.

Supplementary Table S3: Multivariate analysis of survival against various characteristics

	DFS			OS		
	**Hazard ratio**	**95.0% confidence interval**	***p* value**	**Hazard ratio**	**95.0% confidence interval**	***p* value**
**Age**			**0.337**			**0.373**
<40	1			1		
40~59	0.525	0.221~1.247		0.463	0.097~2.208	
≥60	0.709	0.276~1.822		1.084	0.228~5.144	
**surgery**			**0.146**			**0.855**
MRM	1			1		
BCS	0.217	0.028~1.706		1.234	0.129~11.817	
**Histology subtype**			**0.559**			**0.306**
IDC	1			1		
Others	1.863	0.232~14.989		3.123	0.352~27.696	**0.392**
**Grade**			**0.355**			
Low/ Intermediate	1			1		
High	0.688	0.312~1.519		1.819	0.463~7.153	
**P53**			**0.696**			**0.725**
Negative	1			1		
Positive	1.147	0.578~2.276		0.824	0.282~2.414	
**Ki67**			**0.971**			**0.167**
<20%	1			1		
≥20%	0.981	0.358~2.693		0.358	0.083~1.537	
**Stage***			**0.009**			**0.046**
I	1			1		
II	2.538	0.829~7.772		1.334	0.237~7.516	
II	5.120	1.690~15.515		4.697	0.966~22.851	
**TTK expression**			**0.000**			**0.114**
<55	1			1		
≥55	0.204	0.100~0.416		0.398	0.127~1.249	

*Because of multicollinearity between lymphovascular invasion, tumor size, number of positive lymph nodes, and pathological stage, only stage entered Cox regression model in this table.
Abbreviations: MRM modified radical mastectomy, BCS breast-conserving surgery, IDC invasive ductal carcinoma.
